# Impact of Novel and Emerging Nonthermal Technologies on the Functionality and Digestibility of Poultry-Derived Proteins

**DOI:** 10.3390/foods15172971

**Published:** 2026-08-24

**Authors:** Anjana Rajendran, Pilli Pavan Sreehitha, Pavithrakumar Thennarasu, Mariam Shibu Thunjath, Jeyan Arthur Moses

**Affiliations:** Computational Modeling and Nanoscale Processing Unit, Department of Food Process Engineering, National Institute of Food Technology, Entrepreneurship and Management, Thanjavur (NIFTEM-T), Ministry of Food Processing Industries, Government of India, Thanjavur 613005, Tamil Nadu, India

**Keywords:** nonthermal processing, poultry proteins, protein functionality, water holding capacity, emulsification, protein digestibility

## Abstract

Poultry meat and eggs are high-quality protein sources, but thermal processing alters their nutritional and functional attributes. Emerging non-thermal and novel processing methods, such as high-pressure processing, ultrasound, pulsed electric fields, cold plasma, moderate electric fields, magnetic fields, ozone treatment, and pulsed light, are increasingly popular as substitutes. This review examines the effects of nonthermal and novel processing technologies on poultry meat and egg proteins. The review focuses on the interrelationship among protein structure, functionality, and digestibility, with emphasis on the mechanism underlying structural modification, altered intermolecular interactions, and controlled protein unfolding. Under optimized conditions, nonthermal technologies can maintain protein integrity and improve functionality such as water-holding capacity, emulsification, gelation, and tenderness in poultry meat. In egg proteins, non-thermal technologies improve solubility, emulsification, foaming, and gelation through structural modification, intermolecular interactions, and controlled unfolding, thereby exposing enzymatic cleavage sites and enhancing digestibility. Evidence exists for enhanced proteolysis by peptide release and amino acid availability in nonthermal treatments, whereas studies on poultry meat remain limited. This review integrates existing evidence and critical gaps in interpreting structure–functionality–digestibility interrelation, and highlights the potential of nonthermal technologies for producing poultry-derived foods with higher functional quality and nutritional value.

## 1. Introduction

The global population is increasing gradually, reaching 8.2 billion in 2025, and is anticipated to rise to about 10.3 billion by the 2080s. Poultry is an important part of agriculture and a rapidly growing sector. To meet the protein requirements of this growing population, poultry farming plays a crucial role in fulfilling nutritional and protein needs because of its high-quality protein profile. To meet the protein requirements of the growing population, poultry farming plays an important role as a source of high-quality protein. Poultry meat and eggs are mass consumer products, serving as the primary source of affordable protein worldwide. It is a highly perishable food and represents a major source of human foodborne infection. Consumers favor poultry due to its lower price, ease of cooking and nutritional benefits, including a high content of easily absorbed protein, low fat levels, and a rich supply of polyunsaturated fatty acids [1].

Humans started cooking meat thousands of years ago to improve digestibility, modify physicochemical properties, and enhance the flavor and texture by using common cooking methods, including grilling, boiling, pan-frying, roasting, and deep-frying. Conventional thermal processing alters meat protein structure due to extensive denaturation and aggregation, thereby influencing the sensory attributes such as appearance, flavor, and texture, and contributing to both tenderization and toughening. For example, at 58–64 °C, the collagen in meat transitions from a helical/crystalline to a coiled/amorphous form, accompanied by breaking of hydrogen bonds and subsequent relaxation of the fiber. This allows easier shearing during chewing. Also, the incomplete combustion of organic substances during high-temperature cooking can generate heterocyclic aromatic amines, compounds with mutagenic and carcinogenic potential [2]. 

**Figure 1:**
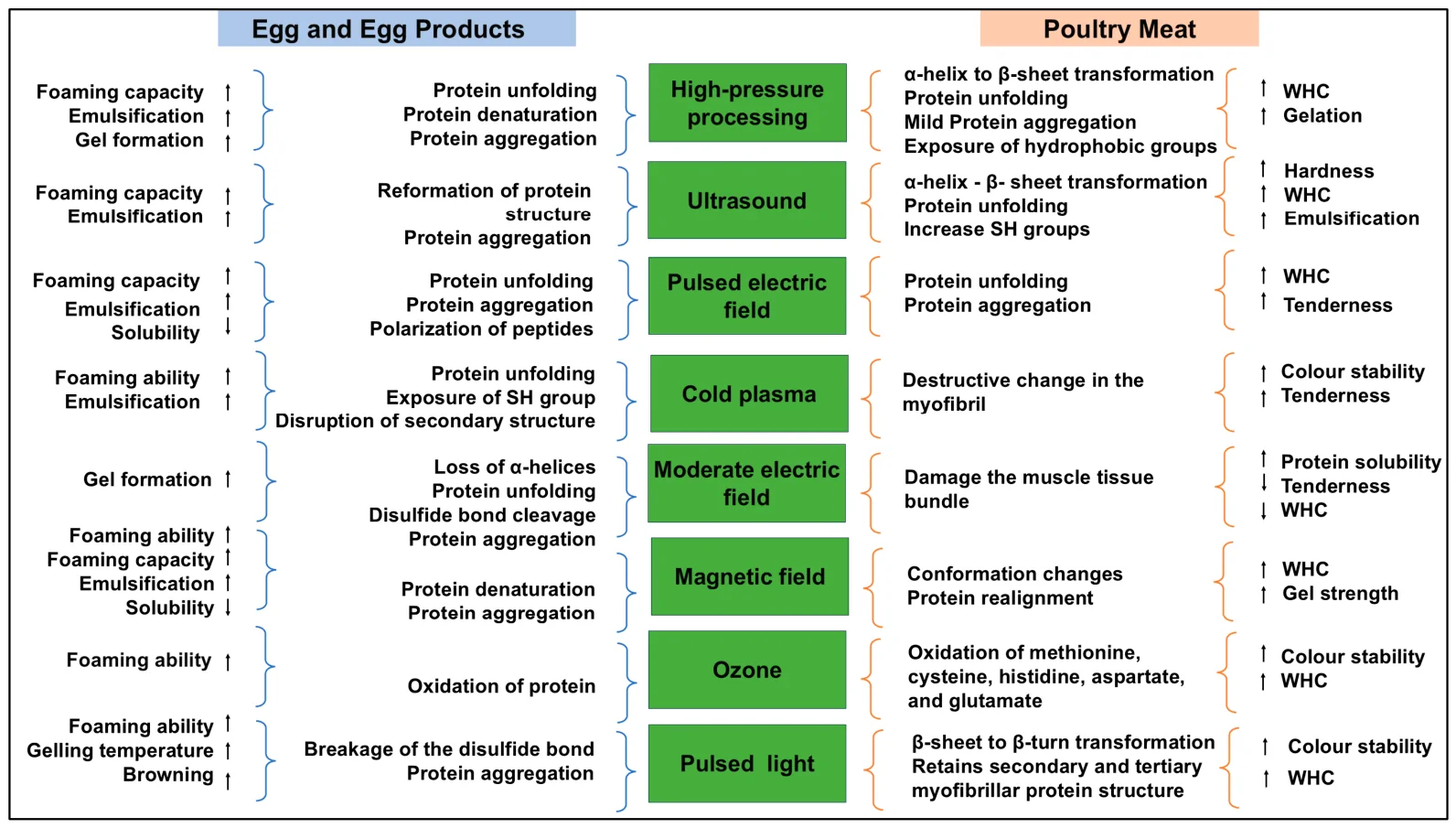
Mechanistic overview of protein structural modification induced by nonthermal novel technologies and their impact on protein functionality. ↑, increasing trend; ↓ decreasing trend; SH, sulfhydryl; WHC, water-holding capacity.

Being an environmentally friendly alternative, nonthermal food processing techniques, such as high-pressure processing (HPP), pulsed electric fields (PEFs), ultrasound, and cold plasma (CP), are innovative and have gained more popularity over the past few decades in food processing, preservation, decontamination, and also in terms of their energy requirement. Under appropriate processing conditions, these techniques can improve the solubility, gelation, water-holding capacity (WHC), emulsification, and foaming properties of meat and eggs, although their effects depend on treatment intensity and processing conditions. But evidence regarding their impact on digestibility remains sparse. The structure and functional features of the protein present in poultry meat are markedly influenced by the processing methods used. Generally, protein unfolding and aggregation induced by nonthermal processing can affect the functionality as well as the digestibility of meat. A mechanistic overview of the relationship between nonthermal processing, protein structural modification and functional outcomes is presented in Figure 1. This gap emphasizes the need for a comprehensive synthesis of current findings and an assessment of how insights from egg protein studies might inform future research on poultry protein digestibility under nonthermal processing conditions [3]. 

## 2. Functionality of Meat Proteins

The physical and chemical characteristics of proteins that influence their behavior during food processing, storage, and cooking are known as protein functionality. Meat protein functional features include their WHC, gelation, and emulsification, and can be profoundly influenced by changes to their physicochemical nature and structure. These properties have a major role in the determination of color, juiciness, texture, binding ability, and sensory attributes of food products, especially in poultry meat and its products [4]. Protein functionality depends on intrinsic factors such as amino acid composition, sequence, molecular size, and charge distribution. Besides extrinsic factors such as temperature, pH, ionic strength, and processing conditions, there are other extrinsic factors that affect protein functionality [5]. WHC is the ability of meat to maintain its inherent water when force is applied and/or processed (e.g., cutting, pressing, grinding, packaging, curing, thermal processing, etc.). Charged and polar groups on myofilament proteins draw and hold water, with an electrostatic swelling force that assists in the immobilization of water in the dense network of proteins. This is associated with net filament charges and binding/retention of polar water molecules. WHC is the net outcome of electrostatic/hydrophilic interactions, osmotic effects through changes in lattice volume, capillary forces in the filament network, and the structural state of the proteins. Overall, these mechanisms can account for why WHC varies as a function of pH, salt, temperature, and processing, although isolating any one dominating mechanism is difficult. Water losses from meat may result in low WHC (excessive loss of moisture), clearly lowering the saleable fresh meat product weight [6]. In addition, the behavior of myofibrillar proteins, such as solubility, cross-bridging, and overall three-dimensional structure, influences the retention of water. Unfolded proteins and the cytoskeletal/intermediate filament structure can disturb adjacent water and make a contribution to WHC independent of merely the globular protein interaction [7]. Controlled nonthermal treatments lead to the formation of a protein network that has a finer pore size and increased polar binding sites, which often show improved WHC. For instance, pressure processing can induce ordered crosslinks without extensive aggregation, which enhances WHC; likewise, ultrasound can increase water binding sites with greater accessibility through partial unfolding. In contrast, over-processing can lead to a reduction of WHC due to the aggregation of protein.

In poultry meat, the major proteins include myofibrillar (50–60%), sarcoplasmic (30–40%), and stromal proteins (10–15%), which collectively determine the overall texture, tenderness, and juiciness of the product. Myofibrillar proteins mainly consist of tropomyosin, myosin, and actin; sarcoplasmic proteins are present in muscle cell fluid, such as myoglobin and glycolytic enzymes, and influence meat color and metabolic activities, while collagen and elastin represent the main stromal proteins. Collagen is largely fibrous and is essential for WHC and emulsification, providing product juiciness and yield, especially in restructured products like sausages [8]. 

Myofibrillar and stromal protein interactions determine the texture and tenderness of poultry meat; enzymatic tenderization and controlled heating application can also enhance tenderness by modifying protein crosslinking. Contrarily, excessive crosslinking of the same may lead to toughness of meat due to morphological changes and altered water retention capability [9]. Texture profiles are a significant quality indicator that can affect the tissue integrity and sensory characteristics of processed meat. Also, light-colored, inadequately gelled poultry meat with low WHC has caused substantial financial loss for poultry farmers [10]. Solubility is considered a prerequisite for other functional behaviors such as emulsification and gelation. Myofibrillar proteins have poor solubility in water but are readily soluble in solutions with appropriate pH and ionic strength. Myofibrillar proteins are insoluble at 0.2 to 0.3 M ionic strength but solubilize at ionic strengths over 0.3 M, and maximum solubilization is achieved at 0.6 M. Greater solubility promotes effective extraction, emulsification, and gel formation in poultry meat products. Conversely, aggregation, denaturation, or enhanced crosslinking reduces solubility, which will impair the quality of meat texture. Solubility relies on the surface distribution of hydrophilic and hydrophobic amino acid groups of protein and the thermodynamic kinetics of protein–water interaction [11,12].

Proteins are amphiphilic molecules that have hydrophobic as well as hydrophilic groups. Provided with adequate input energy, i.e., a mechanical operation like shear, huge fat particles are chopped into fine granules. This process allows protein molecules to adsorb at the fat–water interface, where they organize in a way that the nonpolar groups are embedded in fat (hydrophobic), and the polar groups extend into the aqueous phase (hydrophilic), with the overall decrease in free energy and enhanced stability of the meat emulsion system. The comparative emulsifying activity of muscle proteins is as follows: myosin > actomyosin > sarcoplasmic proteins > actin, in order [13]. Additionally, the protein should be in adequate concentration such that the protein molecules can associate to create a continuous, stable film at the surface of the oil droplet. One of the reasons highly comminuted batters are unstable is because very small droplets have a very great surface area and therefore need more solubilized and extracted protein to create the stabilizing film. Diffusion into the interface, molecular flexibility, and solubility greatly promote emulsification. Adsorption kinetics and emulsion stability are optimized through nonthermal processing that enhances flexibility and surface hydrophobicity. For instance, most PEF and ultrasound treatments strengthen interfacial activity through the creation of surface-active protein species [14]. Therefore, improving the emulsifying properties of these proteins is important for enhancing the quality of meat products and developing new products.

A protein gel may typically be referred to as a viscoelastic body consisting of chains or strands that are crosslinked into a continuous network structure, having the ability to immobilize a high proportion of water. Muscle protein gels are established by a combination of hydrogen bonds, electrostatic interactions, hydrophobic interactions, and disulfide bonds. Gelation is the formation of a gel; in muscle foods, it is the result of unfolding and consequent association of proteins. During the heating of muscle proteins, they unwind or denature after a certain critical temperature is achieved, and later the unwound molecules clump together to develop an ever more viscous solution [15]. Controlled unfolding and reassociation into a three-dimensional network of protein is the primary requirement of gelation [16]. Often, nonthermal processes yield finer viscoelastic properties as compared with conventional thermal processing.

## 3. Functionality of Egg Proteins

A typical whole raw egg, egg white, and egg yolk (along with vitelline membrane) contain 12.50, 10.90, and 15.90 g protein per 100 g, respectively [17]. Egg white consists of approximately 88% water, 11% protein, 0.2% fat, and 0.8% ash. The protein content in egg white is a complex mixture of several protein fractions, which include ovalbumin (around 54%), ovotransferrin (approximately 12–13.6%), ovomucoid (about 11%), ovomucin (roughly 3.5%), and lysozyme (between 3.4% and 3.5%) [18]. Additionally, there are over 30 other protein fractions that contribute to the functional properties of the egg white. Egg proteins have several functional attributes, including foaming, emulsification, heat setting, and acting as a binder. Denaturation of egg proteins, along with their coagulation, is primarily responsible for the functional properties of egg proteins, leading to the formation of a stable matrix. Eggs are popular ingredients in dishes due to their multifunctional properties, such as coagulation, foaming, emulsification, and contributions to a dish’s color and flavor.

Generally, a protein gel network is established through noncovalent crosslinkages like hydrophobic interactions. It is a fact that the heat-induced gelation of proteins usually encompasses an initial protein unfolding and then association reactions, leading to the development of the gel network structure. Ovalbumin is the most abundant protein in egg white and therefore prevails in the gelling behavior. Ovalbumin gelation takes place at 48.5 °C (pH 2.0) to 76.5 °C (pH 9.5). Noncovalent and disulfide bonding stabilize the ovalbumin gel matrix, and the relative contribution of each category of bonding depends on pH, ionic strength, and the heating conditions used to create the gels. A loss of total sulfhydryl (SH) at 80 °C and 95 °C is correlated with gel strength. In contrast, intermolecular disulfide crosslinks are not required for lysozyme gelation but may cause a more stable gel.

When egg whites are beaten vigorously, they create foam that can increase in volume by up to eight times. Ovomucin is primarily responsible for the thick layers of foam. When heated, ovomucin forms insoluble films that help stabilize the foam. Denatured ovalbumin has greater foaming power than native ovalbumin because the proteins adsorb more readily at the air bubble surface. Mild heat treatments of ovalbumin alter the structure from a ‘rigid’ conformational state to an intermediate stable partially unfolded state, also referred to as the ‘molten globule’ state. These altered protein structures showed improved foaming functionality, such as an increased rate of surface tension reduction and an increase in foam stability (liquid drainage reduced) [19]. Four components alternate in the albumen: chalaziferous (or inner thick white), inner thin white, outer thick white, and outer thin layer. The quantity and viscosity of the thick layers of albumen determine the egg’s foaming ability. The principal properties that make a good foaming agent may be summarized as follows: (i) to adsorb quickly at the air-water surface, (ii) to undergo a quick conformational change at the surface, and (iii) to yield a cohesive viscoelastic film through intermolecular interactions [20]. Dishes such as meringues, sponge cakes, angel food cakes, soufflés, fluffy omelets, and various confectioneries depend on the structure provided by these foams to achieve the desired volume and stability [21].

Solubility is the most important physicochemical property of egg protein that can reflect the denaturation and aggregation of protein in the solution. Thus, this property affects other functional properties of proteins, such as foaming and gelation. The application of egg white proteins in the food industry is highly dependent on the foaming properties. Thus, foaming capacity and foaming stability, as the most important functional properties of protein, are important in the baking industry.

A detailed discussion of different nonthermal technologies and their impact on protein functionality is given below. Table 1 and Table 2 summarize the impact of different nonthermal technologies, including HPP, ultrasound, PEF, and CP, etc., on the structural modifications and functionality outcomes of poultry meat proteins and egg proteins, respectively.

## 4. Impact of Novel and Nonthermal Technologies on Protein Functionality

### 4.1. High-Pressure Processing

HPP is a nonthermal processing technique used to inactivate spoilage-causing microorganisms and extend the shelf life of foods while generally preserving several quality attributes. The pressure range of HPP spans from 100 to 600 MPa and operates at a temperature range of 4 to 60 °C. Water is commonly used as the pressure-transmitting medium as it enables relatively uniform hydrostatic pressure transmission throughout the food matrix. Since proteins are pressure-sensitive food components, HPP can substantially alter their conformation and intermolecular interactions, thereby modifying their functional properties [51]. The versatility of meat products depends on the physicochemical and structural characteristics of myofibrillar proteins. Application of pressure will lead to a more compact arrangement of water molecules around electric charges, which increases ionization and has an impact on the pH stability of proteins [22].

HPP induces protein conformation rather than chemical modifications, such as protein unfolding, stretching, and conformational rearrangements, leading to exposure of interior hydrophobic and SH groups, which can enhance protein–protein interactions and surface hydrophobicity. However, it does not break covalent bonds such as peptide or disulfide bonds, thus preserving the primary structure of proteins [52]. SDS-PAGE analysis revealed that HPP induces disulfide crosslinking in myofibrillar proteins, particularly myosin heavy chain and actin. At pressures up to 200 MPa, there was minimal change in myosin heavy chain, but at 300 MPa, a significant loss of myosin heavy chain and the formation of high-molecular-weight polymers were observed. Based on the extent of aggregation of the high-molecular-weight proteins, the protein functionality can be beneficial or detrimental. Generally, moderate aggregation improves gelation and WHC. In contrast, excessive aggregation leads to negative effects on these functionalities. Also, high-molecular-weight proteins are less soluble, thereby reducing solubility, and their excessive crosslinking leads to irreversible insolubilization. Treatment with β-mercaptoethanol largely recovered the lost proteins, indicating that disulfide bonds were responsible for crosslinking and aggregation at higher pressures [53]. Thus, pressure-induced aggregation can have either desirable or undesirable consequences, depending on its extent and the functionality required from the protein system.

**Figure 2:**
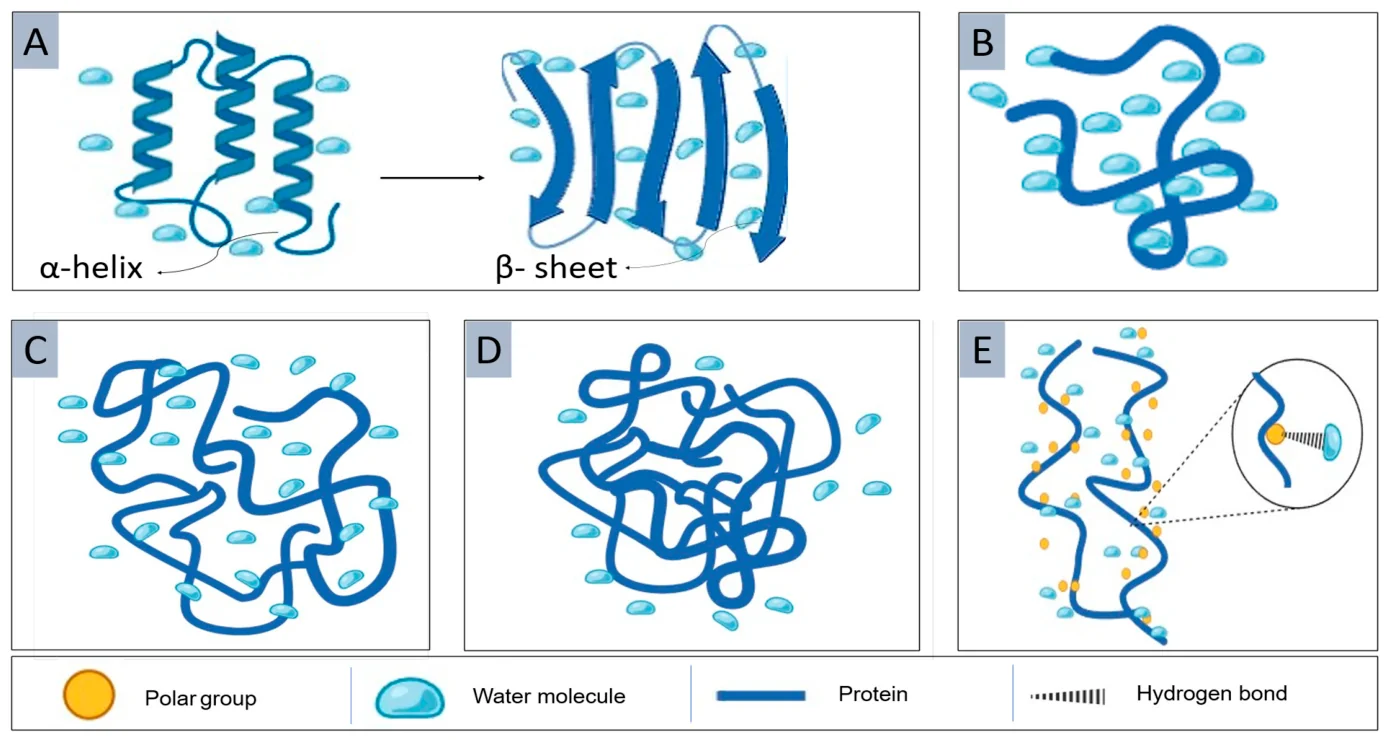
Schematic representation of protein structural modification and the interaction with water molecule and how they affect WHC. (**A**) α-helix to β-sheet transformation; (**B**) partially unfolded proteins with exposed backbone and side-chain polar groups; (**C**) moderate hydrated aggregates that trap water; (**D**) excessive aggregation/compact network that excludes water; and (**E**) unfolded protein with exposed polar groups forming hydrogen bonds to water molecules.

For instance, research on chicken myofibrillar proteins established that 150–200 MPa enhances the water-binding capacity of gels. In contrast, pressure above 300 MPa may reduce WHC due to excessive denaturation and aggregation, causing disruption in the continuity of the gel network and producing larger, less uniform pores that are less effective at retaining water [54]. These findings indicate that the relationship between HPP and WHC is not linear; controlled protein unfolding may improve protein–water interactions, whereas excessive aggregation can impair the structural organization required for water retention. Figure 2 represents the structural modification of the protein and the relation between the WHC.

HPP can also modify protein gelation by altering protein–protein and protein–water interactions. Moderate pressures of approximately 100–300 MPa can promote dissociation of protein complexes and partial unfolding, exposing hydrophobic and sulfhydryl groups that facilitate intermolecular interaction and the formation of a stronger gel network upon heating. In contrast, pressures above 400 MPa may induce rapid and uncontrolled aggregation, resulting in coarse and heterogeneous gel structures with reduced elasticity and firmness [55]. Therefore, the extent of protein unfolding and aggregation must be carefully controlled to obtain the desired gel functionality.

HPP can also influence color and texture of meat, although the magnitude and direction of these changes depend on pressure level, meat species, muscle type and the initial oxidative state of myoglobin. HPP may also alter meat tenderness through pressure-induced changes in the protein matrix [56]. These effects highlight an important practical consideration: processing conditions that improve protein functionality may not produce desired sensory properties.

HPP has also attracted considerable interest for the processing of liquid egg products because pressure-induced protein modification can alter gelation, foaming and emulsifying properties. Pressure processing can lead to the transition of a liquid sample to semi-viscous and promote gel formation, which is suitable for commercial food applications. HPP of whole liquid egg and liquid egg white at 350 to 550 MPa pressure level for 5 to 15 min can lead to partial gel formation, and at a pressure level of 550 MPa, it leads to complete gelatinization. The foam stability is maximum at moderate pressures with shorter times (350–450 MPa for 5 min), giving the highest foam stability in egg white, with foam stability around 1.75 mL, while higher pressures and longer times (e.g., 550 MPa for 5–15 min) reduced foam stability. A pressure of 450 MPa for 5 min can be considered an optimal condition [57]. However, this optimum is application-dependent, as higher pressure or longer treatment can promote extensive gelation and aggregation that may be undesirable when a fluid egg product is required.

HPP (400 MPa for 5 min) is utilized, followed by ultrafiltration to obtain a novel emulsifying component from egg yolk. Emulsions prepared with the ultrafiltration retentate of plasma from HPP-treated granules were more stable against creaming and flocculation compared to those prepared without HPP. Employing moderate pressure (50–400 MPa, 5 min) as a pre-treatment before thermal pasteurization enhanced the emulsifying activity (35–52%) and emulsion stability (41–66%) of egg yolk. Another use of high pressure is its application in hyperbaric storage. Liquid egg yolk storage under hyperbaric pressure at 200 MPa retained its emulsifying activity for 28 days [58].

Overall, HPP can effectively modify the protein functionality through controlled structural changes. However, its effects depend on pressure, treatment time, temperature and protein composition. Moderate pressures may improve functionality, whereas excessive pressure can promote aggregation and reduce solubility. Therefore, process conditions should be optimized according to the desired functionality, while further research is needed to ensure consistent and economically feasible industrial-scale application.

### 4.2. Ultrasound

Ultrasound is an emerging nonthermal technology widely used in the food processing industry. It utilizes sound waves with a frequency >20 kHz. When ultrasound waves travel through a medium, they produce pressure waves that disturb particles, causing the formation of cavities or bubbles. These bubbles grow through successive ultrasound cycles until they become unstable and collapse, generating localized high temperature and pressure [59].

Nowadays, ultrasound is widely used in the meat industry to improve properties such as meat tenderization, emulsification, marination [60], freezing, homogenization, drying, and microorganism inactivation. Ultrasound treatment, along with thermal treatment, is found to be effective for improving the tenderness of meat [61]. Ultrasound is a productive tool for preparing emulsions, especially in normal operating mode, where high emulsion stability is achieved. This is because ultrasound assists in the size reduction of fat globules and improvement in salt diffusion in emulsions, as well as increasing protein solubility. Such emulsions will be acidic due to the production of H^+^ ions, which will bind to NH_2_ groups of the amino acids due to the effect of ultrasound on the structure of the myofibrillar proteins [62].

Ultrasound treatment can be applied to enhance the textural properties of chicken meat; it improves the physical and chemical properties of meat proteins, which can lead to a decrease in the α-helix in the intramuscular protease complex, in addition to a reduction in the viscosity coefficients. A study [63] identified that pulsed ultrasound treatment at 6 min significantly improved the rheological and structural properties of chicken myofibrillar protein without altering the primary protein structure, while disrupting secondary (a decrease in the α-helix and β-sheet proportions and an increase in the β-turn), tertiary, and quaternary structures, as confirmed by Raman analysis. Microstructural analysis showed smaller, more homogeneous protein fragments with a more regular microstructure, likely due to unfolding of chicken myofibrillar protein molecules and exposure of hydrophobic residues and SH groups. In a study of ultrasound processing on chicken breast meat, it was shown that ultrasound can increase the proportion of disordered structures and alter secondary structures, converting α-helices into β-sheets, which results in a looser, more flexible protein network [64]. This will facilitate the movement and uniform distribution of water within meat and result in a higher percentage of immobilized water, thereby improving the WHC. Additionally, the exposure of the hydrophobic group can lead to prominent protein–water interaction, and efficient crosslinking can lead to a more stable gel.

Current application of ultrasound in egg processing is improving functional properties, enhancing food safety and shelf life, and hastening processing techniques. Studies show that ultrasound treatment can enhance the foaming ability of egg white by reforming the protein structure, thereby making it more resilient to adsorb at the air–water interface. Recently, a study [65] reported that foaming of pasteurized liquid egg white was found to be increased by 1.21 times compared to control samples. Also, the shelf life of pasteurized liquid egg white protein can be extended from 30 to 40 days with ultrasound treatment. This is due to the depolymerization effect on pasteurized liquid egg white at optimum ultrasound conditions of 400 W, 60 Hz, 15 min. Additionally, high-intensity ultrasound is effective for enhancing the emulsification and gel-forming properties of egg yolk proteins by modifying their structure and aggregation, which is essential for enhanced quality of the products. Subsequent studies have shown that high-intensity ultrasound treatment of diluted liquid egg yolk (150–210 W) increased viscosity and improved flow behavior with rising temperature (45–60 °C), with an increase in total SH content and maintained enhanced emulsification properties, suggesting practical relevance for egg-yolk product manufacturing and with significant applications in foods such as mayonnaise, ice cream, and cakes to develop texture and stability. In another study, ultrasound treatment at 300 W and 450 W improved the internal quality of fresh egg, such as haugh unit, yolk index, albumen pH, relative whipping capacity, and albumen viscosity, proving that this technique could be effective for maintaining the internal qualities of egg [66]. Thus, ultrasound can improve foaming, emulsifying and rheological properties of egg proteins through controlled structural modification. However, the optimum treatment conditions are application-dependent, and excessive sonication may compromise protein functionality. Industrial application also requires consideration of treatment uniformity and energy efficiency during scale-up.

### 4.3. Pulsed Electric Fields

PEF application on biological materials, such as cells and tissue, causes electroporation, which will increase the membrane permeability [67]. Recently, many studies have been carried out to investigate the effect of PEF on beef protein, egg protein, soybean protein, and whey protein [68]. Meats, when treated with PEF, had their tissue structure altered and their muscle fiber voids and gaps widened, resulting in circular pores and voids in muscle fiber, with their number increasing as the intensity of the electric field applied increased. The voids and gaps will facilitate the easier penetration and movement of water and salt, and expose more enzymatic sites, which will alter the meat protein functionality by protein–water interaction.

In a study by [69], the optimized parameters for PEF treatment of chicken meat are considered to be 18 × 10^2^ kV/m, 8000 Hz, and 3 min, where it could form a heavily crosslinked and dense network structure, which was supported by gel strength. Myofibrillar proteins treated at 18 × 10^2^ kV/m showed higher gel strength due to small particle size and lower water mobility. The application of PEF treatment can enhance the WHC of chicken breast meat, with the combined treatment of calcium chloride and PEF showing a synergistic effect [70].

Low-intensity PEF treatment is more important in chicken meat processing, as it significantly improved WHC (*p* < 0.001), reducing drip by about 28.5% in the course of 4 days of refrigerated storage, without any detrimental effect on protein integrity and functionality [71]. A recent investigation by [69] revealed that PEF processing of chicken meat enhanced the release of umami taste-related amino acids such as glutamic acid and aspartic acid while reducing bitter amino acids. Through protein unfolding and denaturation, the electric field might damage the protein’s double-layer structure and degrade small proteins into peptides and free amino acids. From Table 1, the observations emphasize the importance of determining a suitable PEF intensity level that does not compromise meat quality. PEF has a positive effect on the emulsification of poultry meat proteins. PEF enhances membrane permeability and enables the release of intracellular proteins that are indispensable for the formation of a stable emulsion. Also, moderate physical disruption of protein without denaturation allows superior protein solubilization. This solubilized protein will be available for enhanced interaction with water and lipid molecules, and the protein acts as an emulsifier by stabilizing fat droplets dispersed in the meat matrix [72].

PEF can improve protein functionality through either high-intensity PEF or low-intensity PEF; both have different effects on protein structural modification. High-intensity PEF (25 × 10^2^ kV/m, 200–800 μs) can cause protein aggregation and form insoluble aggregates, resulting in reduced solubility and increased particle size in liquid egg white, which will result in a loss of measurable soluble protein content. Contrarily, low- to moderate-intensity PEF causes less aggregation and minor perturbations in solubility, thereby preventing the loss of soluble protein, along with the preservation of proteins’ native distribution [73]. Under the conditions studied, PEF treatment has a very strong synergistic effect with other processing techniques such as ultra-pasteurization, heat, and additives, as it increases the foaming and emulsifying ability with an enhanced mouthfeel of liquid egg white compared with ultra-pasteurization alone. Hence, PEF (25 × 10^2^ kV/m; 200 kJ/kg), along with heat treatment and additives, is a promising alternative to the industrial ultra-pasteurization of liquid egg white [74]. These findings indicate that PEF intensity is a crucial factor determining whether protein modification is beneficial or detrimental. Low-to-moderate intensity can promote protein solubilization and functional interactions, whereas excessive treatment may cause aggregation and loss of solubility. Therefore, PEF conditions need to be optimized according to the desired protein functionality.

Egg protein is usually hydrolyzed with an enzyme to synthesize antioxidant peptides. In a study, it was revealed that strong PEF treatment could improve the antioxidant activity of peptides prepared by alcalase treatment. This may be justified, as PEF could cause the polarization of the peptide molecule, destroy the noncovalent bonds, and dissociate more subunits, leading to a disorganization of protein structure, which results in the release of more and more free reducing amino acids and small peptides [75]. Although PEF shows potential for improving protein functionality, its industrial application is still influenced by treatment uniformity, process parameters, and the limited evidence available for different food matrices. Further studies are needed to establish optimized conditions and validate its performance at larger processing scales.

### 4.4. Cold Plasma

Plasma is a state of matter consisting of a gas of ions, whereas CP is a plasma that is not in thermodynamic equilibrium and stays at a temperature below pasteurization or sterilization temperature, hence also called nonthermal plasma. The mechanism of inactivation of microbes in food by CP occurs during its generation: gas molecule breakdown forms reactive species such as ultraviolet (UV) photons, free radicals, energetic ions, and highly oxidative atoms or molecules, which are lethal to microbes [76].

Plasma-activated water (PAW) treatment is widely employed in the meat industry for improving the quality attributes of meat protein. PAW is water treated with plasma and is a mixture of highly reactive oxygen and nitrogen species (RONS) [77]. PAW was found to enhance meat protein functionality by reducing protein loss and maintaining muscle fiber integrity during the meat thawing process. A 2 L/min flow rate is the optimum parameter used for PAW for chicken meat and was found not to significantly decrease the WHC as compared to the conventional thawing method [78]. In another study, PAW has been shown to improve the gel strength of chicken myofibrillar protein by promoting the aggregation of protein and also enhancing WHC. A study on duck myofibrillar protein revealed that moderate PAW treatment could improve gelling capacity due to redox reactions with protein molecules. On the other hand, a higher degree of protein oxidation decreases the gelling capacity [79]. These findings indicate that controlled oxidation may improve protein interaction and gel formation, whereas excessive oxidation can impair protein functionality. Thus, the effect of CP depends strongly on treatment intensity and exposure conditions.

A major application of CP is the in-package treatment. Dielectric barrier discharge (DBD)-based in-package CP is a novel nonthermal technology for the destruction of microorganisms and prevention of food spoilage. In DBD, CP is produced between two electrodes at a high potential difference, which are separated by one or more dielectric barriers. A dielectric acts as a stabilizing material, leading to the formation of a large number of micro-discharges when the potential across the gap reaches the breakdown voltage. In a study [80] on boneless skinless breast meat in-package, CP treatment at 60 kV for 60 s was the optimal condition for minimizing appearance changes in raw chicken meat at 4 °C when packed in a barrier film. Ground chicken patties loaded with 1% rosemary extract were treated with in-package DBD-CP at 70 kV for 180 s, showing that it reduced the microbial load, but there were no significant changes in color parameters, except that it decreased the L* values but increased the lipid oxidation [81]. This might be due to the generation of reactive oxygen species such as ozone, hydrogen peroxide, and nitrogen oxides, which can react with lipids and promote oxidation. A similar increase in L* values was also reported [82].

CP treatment could improve the egg protein functionalities by protein unfolding, exposure of the SH group, and increased surface hydrophobicity and disruption of α-helix and β-sheet formation, which resulted in the increased emulsion stability and foaming properties of egg white proteins. Factors such as distance between the plasma source, treatment time, plasma power, and gas composition can affect protein modification [83]. Among many CP treatments, DBD-CP treatment is an effective method for sustaining and enhancing functional attributes, such as increased foaming and emulsion, with enhanced water and oil holding capacities of egg white protein by increasing duration (0, 60, 120, 180, 240, and 300 s) at 40 kV [84]. Another study demonstrated that CP treatments are an effective technique for the efficient generation of amyloid fibrils with improved emulsion stability from ovalbumin [85].

Cold plasma is an emerging food processing technology, and its regulatory status remains dependent on the specific treatment process, food application, treatment conditions, and Jurisdiction. In the United States (US), no general FDA authorization has been established for cold plasma as a food processing technology; regulatory evaluation may instead be required for specific applications under the applicable food safety framework [86]. Despite its potential, CP requires careful control of treatment time, plasma power, gas composition and treatment distance to avoid excessive oxidation. Further studies are also important to establish consistent processing conditions and validate its applicability at an industrial scale.

### 4.5. Ozone Treatment

Ozone is a strong antimicrobial agent against various Gram-positive and Gram-negative pathogens, also called the “allotrope of oxygen”. It is user-friendly since it is economical and can be dissolved in gas and water. Its major advantage is that it leaves no residue on food products since it decomposes to oxygen instantly due to its unstable nature. The Food and Drug Administration (FDA) approved the use of ozone in poultry in 2001. Its mode of action on microorganisms is oxidizing the cell wall and membrane and increasing their permeability, which will eventually lead to cell death. A concentration of 10 to 100 mg/kg ozone gas with a treatment time of 1 to 10 min will effectively inactivate the microbial pathogens in poultry and other meat products [87].

Ozone treatments oxidize the SH groups of amino acids, leading to the generation of shorter peptides from proteins and enzymes. The ability of meat to hold onto water is an important quality. When oxidation happens, it changes the electric charge of proteins. This change causes the proteins to partially unfold and expand. Once proteins are unfolded, they might stick together due to these electric interactions, which can affect how much water they can retain. In terms of muscle texture, myofibrillar proteins play a key role, and the hotspots are myosin heavy chain and actin, which are especially sensitive to oxidation. Common sites for oxidation include methionine, cysteine, histidine, aspartate, and glutamate [88]. The amount of meat protein and WHC is related, as when proteins are denatured, a thin layer may form that helps keep water from escaping. Ozonation of meat can cause the entrapment of water in the gel matrix and thereby improve gel strength by creating a homogeneous microstructure with uniform pore size [89]. The effect of ozonation on protein functionality therefore depends on the extent of oxidation. Controlled oxidation may promote protein unfolding and intermolecular interactions, whereas excessive oxidation can negatively affect protein structure and quality attributes.

Treating turkey meat with ozone for 6 h boosts its cooking yield. Ozone could influence the physicochemical properties of meat and meat products, especially the surface color, due to oxidation of myoglobin, producing metmyoglobin, which will decrease the a* color parameter, resulting in discoloration. Chicken breast samples, when treated with ozone gas at a concentration of 10 mg O_3_/h, showed a decrease in L* and a* with an increase in b* during storage. In contrast, ozone treatment is not recommended for the preservation of duck meat due to the brown color formation, which is undesirable [90].

Ozone treatment is an efficient method to improve the hygienic quality of eggs and effectively reduces the microbial load of *Salmonella typhimurium* at 38.8 mg/kg for 10 to 30 min without affecting foaming ability and foam stability. Ozone concentration and treatment time also determine the egg quality. Whipping capacity and foaming capacity increase with ozone concentration (4 to 6 mg/m^3^) as compared to the control. An ozone concentration of 6 mg/m^3^ maintains the egg yolk pH during storage and stabilizes foam over time. Also, when compared with 2 min and 5 min treatment durations, it is concluded that albumen relative foaming capacity increases as the treatment time increases. A moderate ozonation for 20 min could significantly improve the liquid egg yolk emulsion due to the exposure of the interior hydrophobic group of yolk protein and enhance protein flexibility, leading to a more uniform distribution of egg yolk emulsion droplets [91]. In another study, it was revealed that foaming ability increases when the ozone concentration in aqueous solution is raised. Certain factors, such as amino acid composition, conformation, amino acid sequencing, and surface hydrophobic groups, also affect foam stability. For example, when egg white proteins and whey protein isolates were treated similarly with ozone, the foam stability varied among them. Although egg protein foam stability improved, the improvement was much less than that of egg white protein, which might be due to the difference in the above-mentioned factors [92]. Despite these functional benefits, ozone treatments require careful control of concentration and exposure time to balance microbial inactivation with protein oxidation and undesirable color changes.

### 4.6. Pulsed Light

Pulsed light (PL) is an emerging nonthermal technology that utilizes high-intensity light pulses of short duration on food surfaces for sterilization and decontamination to ensure food safety and quality. In pulsed UV light or high-intensity, broad-spectrum UV light, high-intensity pulses from an inert gas lamp with a wavelength of 100 to 1100 nm are utilized. The mechanism behind microbial inactivation is likely due to the inhibition of DNA replication through dimer formation by photochemical action, an increase in temperature due to photothermal action, and damage to the cell membrane and protein denaturation by photophysical effects. For pulsed UV light, the US FDA permits use of pulsed light for the treatment of food under specified conditions as per the 21 CFR § 179.41 regulation. This specifies that the radiation source should consist of xenon flashlamps emitting broadband radiation over the wavelength range of 200–1100 nm, with a pulse duration not exceeding 2 milliseconds. The treatment is permitted for source microorganism control, and the minimum treatment reasonably required to achieve the intended technical effect should be applied, with a maximum cumulative treatment of 12 J/cm^2^. These provisions apply to the specified pulsed light treatment and conditions of use and should not be interpreted as blanket approval of all pulsed UV technologies or applications in food processing. The surface of food should be smooth with minimal reflectance. Pulse UV has shown antimicrobial action at levels of 1.24 × 10^4^ to 18 ×10^4^ J/m^2^ against *Salmonella enteritis*, *Listeria monocytogenes*, *Staphylococcus aureus*, *Escherichia coli*, etc., and bacterial spores in chicken fillet [93]. A study to determine the effectiveness of LED-UV-based technology at different time points on the quality parameters of chicken meat showed that at wavelengths of 280, 300, and 365 nm, the color and other textural qualities remain unchanged [94]. PL treatment can induce oxidation of myofibrillar proteins and reduce the total SH group. PL treatment will induce a β-sheet to β-turn transformation, which is crucial for protein folding and permits the various elements of secondary structure to form tertiary structure. PL intensity of 300 J/Pulse protects the secondary and tertiary structure of the myofibrillar protein [95]. The effect of PL on protein structure appeared to be treatment-dependent. Moderate exposures may cause limited structural modifications, whereas excessive exposure can promote oxidation, disulfide exchange, and protein aggregation. Therefore, treatment intensity and cumulative energy should be optimized to obtain the desired functional properties without compromising protein integrity. A study conducted by Baptista [96] showed that poultry meat PL-treated samples become lighter, redder, and yellower than control samples. The PL also induced a reduction in aldehyde composition in poultry.

On the other hand, in a study [50], the effect of PL on selected properties of egg white showed browning, aggregate formation by disulfide exchange, and protein backbone cleavage on egg white proteins after PL treatment. The study demonstrated that PL induces higher foam stability and also decreases the gelling temperature of egg white. The changes in these functionalities might be due to the structural changes in the protein. Initially, during PL treatment, the SH content increases due to the breakage of the disulfide bond and the disruption of protein structure, promoting partial protein unfolding. As the pulse increases, a decrease in the SH group is observed, which might be due to intermolecular disulfide bonding between the newly created thiol group and protein radicals, leading to the formation of protein aggregates. The jamming of protein aggregates and the protein fragments in the fluid interstices between bubbles leads to higher foam stability. Although PL shows potential for improving protein functionality alongside microbial control, evidence remains limited for different protein matrices, and further research is needed to establish optimized conditions and assess its industrial-scale applicability.

### 4.7. Magnetic Fields

The use of magnetic fields (MFs) is an emerging nonthermal approach for improving meat qualities by inducing potential conformational changes, thereby improving the functional properties of protein while being less expensive, more environmentally friendly, and without any by-product generation. MF treatment significantly increased the functionality and structural properties of chicken meat by inducing conformational changes such as unfolding of α-helix structures and increased exposure of internal groups, which ultimately resulted in a denser and more ordered gel network, hence improving WHC and gel strength [97]. The Lorentz force of the magnetic field induces currents that will interact with charged particles, such as ions and electrons of microorganisms, and induce magnetoporation, ultimately resulting in leakage of intercellular contents and cell disruption. An operational range of 5 to 50 Tesla (T) is sufficient for microbial inactivation, and the range varies with the kind of food processing [98].

MF-assisted thawing of the meat is widely utilized for enhancing the quality while improving the functional properties of meat. Low-frequency MF thawing hastens the thawing process uniformly by preserving the volatile compounds of the meat, as compared to other conventional thawing methods [99]. Muscle fiber integrity of meat is well maintained during the MF application, leading to less protein denaturation, which effectively reduces water loss, as well as lipid oxidation during thawing. Moreover, the WHC is improved, making the MF a suitable method for thawing meat [100]. Meat stored under a 4 mT static MF at a super-chilled temperature (−3 °C) showed a higher water retention rate and lower drip loss and cooking loss than the control sample. This might be due to the inhibition of myofibrillar denaturation by the magnetic field, which leads to a reduction in myofibrillar protein solubility in phosphate buffer, emulsification capacity, and SH content. This study suggests the potential of MF for meat storage at chilling temperatures [101]. The reported benefits of MF appear to depend on field strength, frequency, exposure time, and food matrix. However, the mechanisms underlying MF-induced protein modification remain less established than those of HPP or ultrasound, and evidence from large-scale application is limited.

MF application is a novel technology for the processing of eggs to enhance food safety and improve functional properties. A recent study by [102] demonstrated that applying an MF of 240 mT for 3 min to chicken egg albumen will reduce the microbial load to 1 log CFU/mL and ensure microbiological safety. Moreover, the egg albumen exhibited a 3% increase in foam stability and a 45% foaming capacity after magnetic field treatment. This research concluded that MF is effective for the pasteurization of egg albumen. Another study applied MF-assisted freezing of egg yolk to a commercial cell-alive system using minimum static MFs and maximum static plus oscillating MFs. MF treatment resulted in protein denaturation and an increase in the SH group, which enhanced the emulsion stability and emulsifying ability. The mechanism behind this phenomenon is that protein denaturation and aggregation during MF treatment leads to protein insolubility, which will negatively affect the achievement of good emulsifying properties. In contrast, the establishment of disulfide bonds at the oil-in-water interface enhanced the emulsion stability [103]. Therefore, although MF shows potential for improving protein functionality, further studies are required to establish reproducible processing conditions and evaluate its feasibility for industrial-scale application.

### 4.8. Moderate Electric Fields

Moderate electric field (MEF) refers to the application of electric fields with a field strength within 1 × 10^2^ to 1 × 10^5^ V/m and a frequency of 50 to 60 kHz, and it also utilizes waves such as sine, square, and sawtooth. Depending on the electric field intensity and strength, molecular changes can be induced in chicken protein, which was proven in a study conducted by [35]. Moreover, it facilitates salt diffusion during marination and can lead to slight changes in pH, which may influence protein functionality, such as water-binding ability and gelation [104]. Compared with other electric field technologies, studies on MEF remain limited, particularly for food proteins, which restricts its current industrial applicability.

MEF has potential applications in the formation of protein gels from animal, plant, fungal, algal, and insect proteins. MEF application causes the unfolding of protein and results in loss of α-helix, disulfide bond cleavage, and aggregation by dipole alignment along the direction of the electric field. Egg protein, which is globular in nature, forms a gel due to the energy produced by intermolecular interactions during heating and denaturation. When heat is applied, it results in the formation of a 3D protein structure due to the dynamic motion of protein peptide chains, which ultimately leads to amino acid bonding with other proteins. MEF can lead to structural modification in egg proteins, which may induce changes in foaming properties, as supported by a study [105] where pretreatment by MEF alters the emulsifying and foaming ability of protein by promoting lipid interaction and improving surface activity. Although MEF shows potential for modifying protein functionality, future research is needed to clarify the relationship between processing conditions, protein structural changes and functional properties and to validate its performance at industrial scale.

### 4.9. Comparative Status and Research Need of Nonthermal Technologies

The nonthermal processes discussed in this review vary based on their degree of technological development and industrial validation. HPP is one of the more developed and established processes, whereas ultrasound and PEF, on the other hand, show great promise but still require further optimization and scaling. CP, PL, and MEF are still emerging technologies with little validation in proteinaceous foods such as chicken meat products. Further research should be directed at optimizing process parameters, increasing scalability and uniformity of treatment, and establishing a clear connection between processing-related structural modifications, protein functionality and digestibility.

## 5. Effects of Nonthermal and Novel Processing Technologies on Protein Digestibility

Digestibility is a key parameter for assessing dietary protein quality and bioavailability. It assesses the amount of protein digested and absorbed from food. It predicts the availability of amino acids following protein hydrolysis occurring in the gastrointestinal tract. Since the body can only obtain essential amino acids from the diet, the digestibility of protein sources governs their contribution to nutrition and muscle protein synthesis [106]. Various methods, including static and dynamic in vitro models, cell cultures, and in vivo studies in animals and humans, are used to evaluate protein digestibility. While in vivo methods assess digestibility, bioaccessibility, and bioavailability, they are costly, time-consuming, and raise ethical concerns [107].

The digestibility of protein is influenced by its structural characteristics, which determine the accessibility of peptide bonds to digestive enzymes. Food processing can modify these structural characteristics and consequently alter protein digestibility, peptide release, and the subsequent bioaccessibility of digestion products. Therefore, understanding the relationship between protein structure and digestion is essential for evaluating the nutritional implications of nonthermal technologies.

Protein digestion involves the sequential hydrolysis of dietary proteins into smaller peptides and amino acids in the gastrointestinal tract. Although limited protein digestion may occur in the oral cavity, substantial hydrolysis occurs in the stomach, where hydrochloric acid promotes protein denaturation and facilitates conversion of pepsinogen into its active form, pepsin. The enzyme pepsin cleaves peptide bonds and converts intact proteins into smaller peptides. In the small intestine, pancreatic proteases, including trypsin, chymotrypsin and carboxypeptidases, further hydrolyze these peptides. Brush-border peptidases produce small peptides, and amino acids must be rapidly and effectively absorbed from the intestinal lumen by the enterocytes of the small intestine [108].

Bioaccessibility is defined as the proportion of nutrients or digestion products released from the food matrix during digestion in the gut and becoming accessible to available for absorption. During protein digestion, it is related to the release and solubilization of peptides and amino acids formed as a result of gastrointestinal hydrolysis. Protein processing may affect the bioaccessibility of proteins to digestive enzymes. For example, protein modifications, including partial denaturation, may lead to exposure of proteolytic sites, allowing peptides to be released. However, excessive aggregation and crosslinking of proteins may hinder the action of digestive enzymes and prevent the formation of digestive products. Peptide liberation is the term used for the process that creates smaller peptides by breaking down whole proteins through gastrointestinal proteolysis, whereby the amount, size and even sequence of the peptides created affect their further absorption and activity [108].

**Figure 3:**
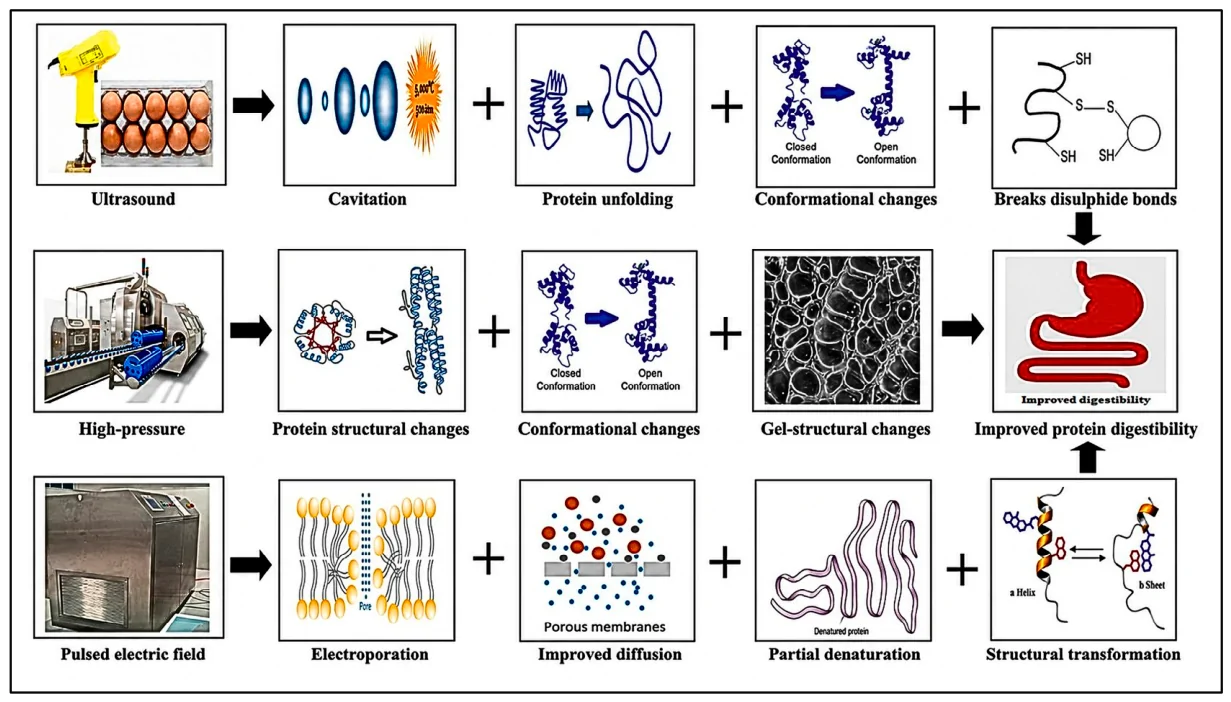
Mechanisms of processing technologies for improving the digestibility of egg proteins (adapted from [112]).

Poultry meat and eggs are good sources of high-quality protein. Processing can alter protein structure and consequently influence susceptibility to gastrointestinal proteolysis. Conformational alterations, including partial unfolding and exposure of enzyme-accessible sites, may result from processing technologies, such as HPP [52], PEF [109], and ultrasound [110]. These structural changes may lead to enhanced accessibility to cleavage sites by digestive proteases [111]. Partial unfolding of protein may expose previously buried peptide bonds and hydrophobic or polar groups, thereby facilitating enzymatic hydrolysis. Therefore, the degree and type of structural alteration will determine whether or not it improves digestibility. The mechanisms by which different processing technologies enhance digestibility of egg proteins are illustrated in Figure 3.

Direct studies investigating the impact of nonthermal and novel emerging technologies on the digestibility of poultry meat are scarce. Bhat et al. [113] reported that although many studies have investigated the effect of thermal techniques, other techniques such as curing, drying, marination, etc. influence the digestibility of muscle proteins. Kim et al. [114] used a two-step in vitro enzymatic assay that mimicked intestinal (pancreatin) and gastric (pepsin) digestion to examine the effects of thermal and nonthermal techniques on the in vitro ileal disappearance of crude protein in chicken meat. Protein digestibility was significantly decreased by thermal processing at 70–121 °C because of heat-induced denaturation, oxidation, and aggregation. On the other hand, crude protein digestibility was not considerably impacted by nonthermal processing techniques such as HPP (500 MPa, up to 7 min), UV-LED radiation (15.6 W/m^2^, up to 120 min), electron-beam irradiation (up to 10 k Gy), or gamma-ray irradiation (up to 10 k Gy). These findings suggest that, under the conditions investigated, the evaluated nonthermal treatment had limited effects on the protein digestibility of chicken meat compared with the reductions observed following thermal processing. Thus, nonthermal treatments may offer potential for microbial safety while minimizing changes in protein digestibility; however, further studies are required to establish whether this effect is consistent across poultry species, muscle types and processing conditions.

H. Li et al. [115] investigated the impact of ultrasound on the protein digestibility of chicken gizzards, which have a tough texture and are not easily digested by the human body. They reported that protein digestibility increased in chicken gizzards that were pretreated with ultrasound (500 W, 30 kHz, for 30 min) compared to those that were untreated. This might be due to the expansion of protein structure induced by ultrasound pretreatment, which increases the cleavage sites of digestive enzymes. However, evidence from egg protein systems, which represent distinct poultry-derived matrices, indicates that nonthermal processes can alter protein digestibility through changes in protein conformation [112]. These findings provide useful mechanistic insights but cannot be directly extrapolated to poultry muscle.

Current studies on the impact of nonthermal treatments on the protein digestibility of poultry meat are quite limited, and the available evidence is insufficient to draw strong conclusions regarding their overall effects. Therefore, definitive conclusions about the ability of these technologies to improve poultry-meat protein digestibility cannot yet be drawn, highlighting a significant research gap that needs further investigation. In contrast, the impact of nonthermal treatments on the digestibility of egg proteins, which are also derived from poultry, has been the subject of numerous studies. By causing changes in the microstructure or structural characteristics of egg proteins, these processing technologies mediate the functionality and quality of eggs. This can impact the digestibility of the eggs and their behavior during digestion in the gastrointestinal tract.

Table 3 summarizes studies investigating the effect of nonthermal technologies on the structural characteristics and digestibility of egg proteins. Although egg proteins are derived from poultry, these findings represent evidence from an egg protein matrix and should not be considered direct evidence of protein digestibility in poultry muscle. The reported findings suggest that controlled unfolding and conformational rearrangements can enhance enzymatic hydrolysis and protein digestibility, whereas excessive aggregation may limit enzyme accessibility. Whether these structural–digestibility relationships occur similarly in poultry muscle remains to be established.

Previous studies on liquid egg, egg yolk, and egg white have mainly concentrated on heating kinetics, protein denaturation, gelation, and functional properties such as solubility, foaming capacity, water-holding ability [121], and color parameters, with comparatively limited assessment of gastrointestinal proteolysis. Future studies should aim to address this gap by utilizing standardized in vitro digestion protocols, such as the INFOGEST method or simplified assays involving pepsin and pancreatin.

## 6. Conclusions

HPP, ultrasound, PEF, CP, MEF, MF, ozone treatment, and PL represent promising nonthermal and emerging processing technologies for modifying the structural and functional properties of poultry-derived proteins. Under optimized processing conditions, controlled structural modifications can improve functionalities such as WHC, gelation, emulsification, foaming, and overall texture in both meat and egg proteins, while helping maintain color and sensory quality upon optimal application. 

Although the functional effects of these technologies are increasingly well-documented, particularly for egg systems, evidence of digestibility improvements in poultry meats remains limited. Current findings indicate that nonthermal treatments may limit excessive aggregation and oxidation while enhancing protease accessibility through controlled modification of protein structure. However, the scarcity of studies using standardized in vitro digestion methods, like INFOGEST, restricts the ability to draw definitive conclusions.

Future research should focus on clarifying structure–digestibility relationships using standardized digestion models and validating optimized processing conditions for practical applications.

## Figures and Tables

**Table 1:** Impact of different nonthermal and novel technologies on the structural modifications and functionality of poultry meat proteins.

Technology	Key Mechanism	Poultry Protein Studied	Processing Parameters	Protein Structural Changes	Effect on Functionality	References
HPP	Le Chatelier’s principle	Chicken breast	450 MPa, 10 min	Protein unfolding, pigment deterioration, and structural changes	Brighter L* values and less redness a*	[22]
		Reduced-sodium chicken myofibrillar protein gel containing calcium chloride	150–200 MPa, 10 min	Solubilization of myosin heavy chain and actin, a decrease in protein aggregation, and exposure of tyrosine and tryptophan residues resulting from the unfolding of the protein tertiary structure	Elevated WHC	[23]
			250–300 MPa, 10 min	Protein aggregation, disulfide crosslinking, and hydrophobic arrangements	Decrease in WHC	
		Chicken breast	450 MPa, 10 min	Protein denaturation and aggregation	Increase toughness	[22]
		Chicken meat	200 MPa, 10 min	Protein denaturation	Optimized myofibrillar protein gelation properties Improved gel strength	[24]
Ultrasound	Produces pressure waves that disturb particles, causing the formation of cavities or bubbles; shear act	Chicken breast meat	40 kHz, 300 W, 20 min	Formation of compact microstructure with protein filaments and strands	Improved texture and WHC	[25]
			40 kHz, 300 W, 40 min	Protein aggregation and cavitation	No improvement or negatively affected the WHC and textural properties	
		Goose meat	50 kHz, 25 °C, 5 min	Closer and irregular modification of the myofibrillar protein	Increased hardness value, increased emulsification capacity	[26]
		Chicken meat	20 kHz 15.6 × 10^4^ W/m^2^, 5 min	Transformation of α-helix to β-sheet, reduced aggregation of myosin	Increased WHC	[27]
		Turkey meat	35 kHz, 215 W, 40 min	Protein unfolding increased the reactive SH group	Increased WHC	[28]
PEF	Electroporation: an increase in membrane permeation	Pale soft exudative chicken myofibrillar protein	18 kV for 30 ns	Changes in spatial structure by H-bond formation and exposure of the hydrophobic group	Increased WHC	[29]
		Duck meat	3 kV, 30 s	Myofibril disruption and vacuolization	Improved texture, improved WHC, reduced L* value	[30]
		Chicken breast meat	120 pulses,1000 V, 50 μs pulse width, 1 Hz	Increased porosity by unfolding or denaturation	Increased water diffusion	[31]
		Pale soft exudative-like chicken meat	18 × 10^2^ kV/m, 800 Hz, 3 min	Ionization of charged residues, alteration of secondary and tertiary structure	Improved protein solubility	[32]
		Goose meat	15 × 10^2^ kV/m, 200 Hz, 10 min	Partial unfolding and aggregation, protonation of protein	Improved tenderness and WHC	[33]
		Pekin duck meat	2 × 10^2^ kV/m 50 Hz, 2000 μs pulse width, 12 °C	Lower protein aggregation	Increased WHC, uniform gel structure	[4]
CP	Generation of reactive oxygen species and UV photons, free radicals	Chicken breast fillets	(Argon-based DBD-CP) 20 kV, 3–5 min	More layer formation in myofibrillar proteins with a wrinkled surface	Decreased shear force, improved tenderness	[34]
Moderate electric field	Utilizes waves such as sine, square, and sawtooth	Chicken meat	50 Hz sine wave 7 min 33 s	Denaturation of sarcoplasmic proteins linked to myofibrillar proteins	Reduced water-soluble protein content Reduced WHC and affected texture (stiff)	[35]
MF	Induce conformational change	Emulsified meat	3 m T DC-MF, 15 min	Realignment and aggregation of proteins, uniform pore size	Increased WHC, improved gelation, textural, and rheological behavior	[36]
Ozone treatment	Oxidation increases permeability	Turkey meat	Concentration of O_3_ = 1 × 10^−2^ kg/m^3^, 8 h	Oxidation of myosin heavy chain, alteration of protein structure	Improved WHC and cooking yield	[37]

HPP, high-pressure processing; WHC, water-holding capacity; PEF, pulsed electric field; CP, cold plasma; DBD-CP, dielectric barrier discharge–cold plasma; UV, ultraviolet; MF, magnetic field; DC-MF, direct current magnetic field; SH, sulfhydryl.

**Table 2:** Impact of different nonthermal and novel technologies on the structural modifications and functionality of egg proteins.

Technology	Egg Protein/Sample Studied	Processing Parameters	Protein Structural Changes	Effect on Functionality	References
HPP	Egg white pudding	500 MPa, 15 min	Altered β-sheet and β-turns structure	Increased hardness, increased microporosity, and lowered syneresis	[38]
Ultrasound	Egg white proteins	20 kHz, 15 min	Conformational changes in globular proteins	Increased solubility and emulsification	[39]
	Egg white protein	0.4 W/mL, 10 min; followed by heating at 72 °C for 10 min	Modified protein surface hydrophobicity and flexibility	Increased foaming stability and emulsification	[40]
	Egg white protein	20/40 kHz, 30 min	Partial protein unfolding and exposure of hydrophobic and free SH groups	Improved the solubility and foaming properties of egg white protein, and improved the WHC of egg white protein gel	[41]
	Egg yolk protein	20 kHz, 300 W, 10 min	Enhanced zeta potential and increased free SH group of egg yolk proteins; aggregation of low-density lipoprotein	Improve the emulsifying, foaming, and gel properties of egg yolk	[42]
PEF	Ovomucin-depleted egg white	1.8 kV, 300 Hz, 20 μs pulse width, pH 5	Increased protein aggregation	Decreased turbidity	[43]
High-intensity PEF	Liquid whole egg	19 × 10^2^ kV/m, 6 µs, 250 Hz	Slight protein aggregation and denaturation	Minimal impact on the foaming capacity when compared to the untreated sample, and increased WHC	[44]
CP	Egg white proteins	2.2 kHz, 4.2 kV, 30 min	Protein unfolding, exposure of the SH group, and increased surface hydrophobicity	Increased emulsion stability and foaming properties	[45]
Magnetic induction electric field	Egg white proteins	600 V, 50 kHz, 3 min	Changing the protein charge distribution, increasing the intermolecular electrostatic repulsion, promoting the exposure of hydrophobic groups, and increasing the porous structure	Improved foaming ability	[46]
MF-assisted freezing	Egg white	1.66 mT, 6 Hz, 75 min	Protein denaturation and aggregation	Mild decrease in foaming ability	[47]
	Egg yolk	1.52 mT, 6 Hz oscillating MF, 75 min	Protein denaturation and aggregation	Increased L* value, higher free SH group, decreased emulsification value, and no significant change in emulsification	[48]
UV processing	Egg white	10.6 kJ/m^2^, 35.4 W/m^2^, 5 min	Protein aggregates, jamming in the fluid interstices between bubbles and/or due to the higher viscosity of the aqueous phase	Higher foam stability, no significant change in gelling behavior	[49]
PL	Egg white	1.75 × 10^−4^ m^2^, 0.50 μs pulse interval, 25 °C	This effect was attributed to jamming of protein aggregates and fragments in the fluid interstices between bubbles	Higher foam stability, no significant change in gel strength	[50]

HPP, high-pressure processing; WHC, water-holding capacity; PEF, pulsed electric field; CP, cold plasma; UV, ultraviolet; MF, magnetic field; SH, sulfhydryl; PL, pulsed light.

**Table 3:** Impact of different nonthermal technologies on the structural modifications and digestibility outcomes of egg proteins.

Technology	Protein/Sample	Parameters	Structural Changes	Digestive Model	Digestibility Outcome	References
HPP	Ovalbumin and egg white	400 MPa, 30 min	Partial unfolding, and increased susceptibility to proteolysis (pepsin + Corolase PP)	In vitro gastric and duodenal digestion	Improved digestibility due to greater accessibility of cleavage sites	[116]
	Egg white protein	550 MPa, 15 min	Partial unfolding of protein	Trypsin hydrolysis	Increased amino acid hydrolysis	[117]
	Whole egg white	800 MPa, 9 °C, 5 min	Extensive denaturation, structural modifications, gel formation and aggregation	In vitro pepsin digestion and proteomic methods	Greater peptide bond exposure and susceptibility to pepsin digestion, improved digestibility	[118]
Ultrasound	Egg white proteins (ovalbumin, ovotransferrin, lysozyme)	20 kHz, 300 W, 15 min	Increase in surface hydrophobicity and SH group exposure due to disulfide bond breakage, and conformational rearrangement	In vitro enzymatic assay with alcalase	Exposure of buried sites, increased susceptibility to proteolysis (alcalase digestion); the degree of hydrolysis increased	[39]
	Egg white protein	0.4 W/mL, 10 min	Cavitation-induced disruption of compact protein structures and digestibility	In vitro gastrointestinal digestion	Improved digestibility for proteins >30 k Da; reduced avidin activity	[40]
PEF	Ovomucin-depleted egg white	1.4 × 10^2^ to 1.8 × 10^2^ kV/m, 259–695 kJ/kg, 20 µs, 300 Hz	Exposure of buried SH and hydrophobic groups/S-S bond cleavage and protein aggregation	In vitro gastrointestinal digestion	Enhanced enzymatic hydrolysis of ovalbumin and enhanced protein digestibility	[119]
CP	Egg white protein	110 kV, 50 Hz, 30 min	Reduced α-helix content, increased hydrophobic residue exposure, and protein unfolding	INFOGEST based in vitro digestion	Enhanced intestinal phase digestibility through increased protease site exposure	[120]

HPP, high-pressure processing; PEF, pulsed electric field; CP, cold plasma; SH, sulfhydryl.

## Data Availability

No new data were created or analyzed in this study. Data sharing is not applicable to this article.
